# Internet Databases of the Properties, Enzymatic Reactions, and Metabolism of Small Molecules—Search Options and Applications in Food Science

**DOI:** 10.3390/ijms17122039

**Published:** 2016-12-06

**Authors:** Piotr Minkiewicz, Małgorzata Darewicz, Anna Iwaniak, Justyna Bucholska, Piotr Starowicz, Emilia Czyrko

**Affiliations:** Department of Food Biochemistry, University of Warmia and Mazury in Olsztyn, Plac Cieszyński 1, 10-726 Olsztyn-Kortowo, Poland; darewicz@uwm.edu.pl (M.D.); ami@uwm.edu.pl (A.I.); justyna.bucholska@uwm.edu.pl (J.B.); pointer86@wp.pl (P.S.); formerly@wp.pl (E.C.)

**Keywords:** bioinformatics, biological activity, chemical information, database screening, education, food informatics, structure search, similarity search, text search

## Abstract

Internet databases of small molecules, their enzymatic reactions, and metabolism have emerged as useful tools in food science. Database searching is also introduced as part of chemistry or enzymology courses for food technology students. Such resources support the search for information about single compounds and facilitate the introduction of secondary analyses of large datasets. Information can be retrieved from databases by searching for the compound name or structure, annotating with the help of chemical codes or drawn using molecule editing software. Data mining options may be enhanced by navigating through a network of links and cross-links between databases. Exemplary databases reviewed in this article belong to two classes: tools concerning small molecules (including general and specialized databases annotating food components) and tools annotating enzymes and metabolism. Some problems associated with database application are also discussed. Data summarized in computer databases may be used for calculation of daily intake of bioactive compounds, prediction of metabolism of food components, and their biological activity as well as for prediction of interactions between food component and drugs.

## 1. Introduction

Recent years have witnessed an unprecedented increase in the amount of data relating to the physicochemical properties, biological activity, enzyme-catalyzed reactions, and metabolism of chemical compounds, including food components. The Era of Big Data creates new challenges in science and education. The storage, search, and retrieval of Big Data require highly specialized and dedicated methods [1].

Databases of chemical compounds are widely used in the chemical, biological, and medical sciences. Their role in food science has been equated with that of traditional sources of information such as journals and books [2,3]. Traditional data sources still prevail in education, but online databases and programs may serve as auxiliary tools that create access to detailed information about specific compounds. Several reviews of small molecule databases have been recently published [4,5,6,7,8]. These reviews include e.g., databases of phytochemicals important from the point of view of food science [4], compounds relevant for nutrition science [5], medical sciences [6], and research on foods of animal origin [7]. Reviews include both free-accessible and commercial resources [8]. Databases from all categories from genes and genomes to small molecules are described [6,7]. Reviews also discuss the strong and weak points of particular databases and future needs. Recent reviews mainly focus on databases as research tools. The database search opportunities and applicability in education are not so extensively described. Results of research involving applications of databases in food and nutrition sciences were published mainly in 2014 or later and could not be included in the cited reviews.

A major advantage of computer databases over books and journals is that they can be regularly updated. Computer databases also provide extensive search options. The use of specialized databases facilitates data mining based on secondary analyses of large datasets which are highly useful in medical and nutrition sciences [9,10]. Compound datasets can also be developed for in silico analyses based on both computer databases and traditional resources. A dataset of DNA inhibitors of methyltransferases applied by Fernández-de Gortari and Medina-Franco [11] is an example of this approach to data mining. The advantages mentioned above make computer databases useful tools in education. They provide rapid access to up-to-date and high-quality information, although users may require training in use of molecular structures written using machine readable codes and drawings. Application of databases follows recent worldwide trends in the use of information resources. In the contemporary world, university students become familiar with computers and the Internet from the childhood. The Internet is often a first-choice tool for finding diverse types of information, e.g., in the field of chemistry and biochemistry. Users who rely on online sources of information face two problems. The first is the quality of information found in anonymous resources, and the second is the use of appropriate queries in popular search engines such as Google™. Specialized databases provide more reliable information and more search opportunities than popular websites. The application of online resources in chemical education was recently described in a special issue of the *Journal of Chemical Education* [12,13,14]. Databases of small molecules are also recommended as educational tools for study fields other than chemistry, including food technology and human nutrition [15].

Bioinformatics methods and tools are recommended for use in the research concerning various approaches associated with food. Holton et al. [2] listed five areas in food science that have benefitted from the use of bioinformatics: “omics” technologies, bioactive peptides, food quality, taste and safety, allergen detection, and food composition databases. The first, third, and fifth areas of interest involve databases of small molecules. The application of bioinformatics in research into bioactive peptides can be expanded to include the biological activity of all classes of small molecules. Bioinformatics tools can also play an important role in food science education. The classic approach to bioinformatics, which focuses on genes and biomacromolecules [16], is not widely applied in agricultural sciences, including food science [17]. Tools designed for research into small molecules, such as databases, may also support education in the fields of food technology and human nutrition.

Individual databases and their content have been extensively reviewed in recent articles [4,5,6,7,8]. Databases of small molecules offer similar options for searching compounds of interest. This review will focus on search options in various databases, navigation through database networks, and examples of secondary analyses of datasets in food sciences. The databases and other tools cited in this publication are summarized in Table 1.

## 2. Chemical and Biological Approach

In in silico studies, the properties of small molecules can be analyzed with the use of bioinformatics and cheminformatics tools. According to the simplest definition, bioinformatics is the application of computer-aided methods for solving biological problems, whereas in cheminformatics, computer-aided methods are used to solve chemical problems. Detailed definitions of cheminformatics have been recently proposed [52]. Cheminformatics methods focus mainly on drug design and the biological activity of small molecules, therefore they should be considered in tandem with bioinformatics tools.

In in silico analyses, the relationships between the structure of a compound and its physical (melting point, hydrophobicity, hydrophilicity) and chemical properties (acid or base, oxidizing or reducing properties) belong to the realm of cheminformatics. The physicochemical properties of food components may affect their mutual interactions, the structure of the end product, and the behavior of ingredients during industrial or small-scale processing (functional properties) [53,54,55]. Chemical properties also affect the biological activity of food compounds. Compounds with reducing properties act as antioxidants, and they play an important role in functional foods [56]. Food flavoring should be analyzed in view of the chemical composition of foods [55,57].

**Figure 1:**
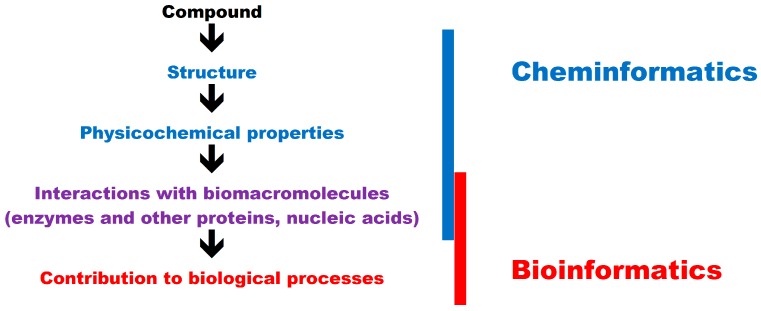
Chemical and biological approaches to in silico research into small molecules.

The overlap between bioinformatics and cheminformatics is presented in Figure 1.

The biological activity of small molecules generally involves interactions with biomacromolecules. Proteins, in particular enzymes, are the most common targets for bioactive compounds. Food components may be substrates, products, inhibitors, or activators of enzymes. A robust knowledge of enzymatic reactions in different compounds is essential in food technology and biotechnology [58]. Enzymatic reactions are part of human metabolic pathways, and they are of great interest for nutritional scientists. Small molecules also interact with proteins involved in cellular signaling as well as other biomacromolecules such as nucleic acids. The interactions between small molecules and biomacromolecules are investigated with the use of software that relies on both bioinformatics and cheminformatics tools [59,60]. Discrimination between areas of interests of cheminformatics reflects tradition. Cheminformatics was focused on properties of small molecules whereas bioinformatics was focused on biomacromolecules. Interactions between these compounds, studied in silico, belong thus to both approaches.

Bioinformatics and cheminformatics use different languages [7]. The language of biology is composed of nucleotide and amino acid sequences. Nucleotide sequences are written in single-letter code, whereas amino acid sequences are labeled with single-letter or multiple-letter code. Chemists use their own codes to describe small molecules: the Simplified Molecular Input Line Entry System (SMILES) [61] and the International Chemical Identifier (InChI) [62]. The language of biology has been designed to describe large molecules composed of repeatable units (nucleotides, amino acids). This language supports the presentation of information concerning large molecules in compact form. The language of chemistry creates opportunities for describing the detailed structure of molecules belonging to all classes of compounds. On the other hand, chemical codes require many more characters for structure annotation than biological ones. This comparison may be illustrated by the example presented below. Some compounds, such as peptides, may be annotated using both chemical and biological languages [63]. For instance, peptide l-prolyl-l-proline may be described with a single-letter or a multiple-letter code of its (PP and Pro-Pro, respectively), the SMILES code (C1C[C@@H](C(=O)N2CCC[C@H]2C(=O)O)NC1) or the InChI code (InChI = 1S/C10H16N2O3/c13-9(7-3-1-5-11-7)12-6-2-4-8(12)10(14)15/h7-8,11H,1-6H2,(H,14,15)/t7-,8-/m0/s1).

Translation of molecular structure annotation between biological and chemical language still provides challenges, although some programs serving this purpose are available. OpenBabel is a freely available, downloadable program designed for translation molecular structures from one code into another [40]. Sequences of oligonucleotides written in FASTA format may be translated into chemical codes using this program. Peptide sequences annotated in a single-letter code (FASTA format) may also be translated into SMILES or InChI using a recent version (2.4.1) of the OpenBabel program, but this option is a weak point of the program. The origin of the problem with this program is the fact that amino acids and nucleotide residues are encoded using the same symbols in single-letter code (e.g., the symbol “A” means adenine or alanine in a nucleotide or amino acid sequence, respectively). OpenBabel considers nucleotide sequences as default. It translates peptide sequences only if they contain symbols absent from single-letter nucleotide code (such as “F”—the symbol for phenylalanine). Translation of peptide sequences should include addition phenylalanine symbols to the sequence (e.g., at the C-terminus), translation into SMILES code using OpenBabel (provided by international consortium), displaying the resulting peptide structure using a molecular editor, removal of additional phenylalanine, and translation of the final structure into SMILES [19]. The above procedure is not described in the OpenBabel user manual. Sugar molecules are described with the use of special codes such as the Web3 Unique Representation of Carbohydrate Structures (WURCS) (WURCS Working Group, Japan). Specific sugar codes provide opportunity for more compact annotation of sugar molecules than universal chemical codes. For instance, sialic acid (PubChem CID 906, ChemSpider ID 21232675) is written in WURCS code as WURCS = 1.0/1,0/[a2d21122h|2,6|2*O|5*NCC/3=O], whereas in SMILES code it is [C@@](O)(C(O)=O)1O[C@]([H])([C@H]([C@@H](O)CO)O)[C@@H](NC(=O)C)[C@H](O)C1. WURCS is also the name of the program that has been designed to translate sugar structures from one code into another [51]. The program utilizes specific codes for carbohydrates but does not provide an option for direct translation between specific sugar codes (e.g., WURCS) and universal chemical codes (e.g., SMILES). The program contains a molecular editor that enables the drawing of the sugar structure and conversion into SMILES or WURCS code. Acylglycerols are also annotated in a specific code in lipid databases, such as LipidMaps (provided by international consortium) [34]. For instance 1,2,3-trihexadecanoyl-sn-glycerol (PubChem CID 11147; ChemSpider ID 10674) is annotated in this code as TG(16:0/16:0/16:0) (its SMILES code is CCCCCCCCCCCCCCCC(=O)OCC(COC(=O)CCCCCCCCCCCCCCC)OC(=O)CCCCCCCCCCCCCCC). LipdMaps database utilizes also InChI code and InChIKeys. Significant progress is necessary to break the language barrier and develop user-friendly tools for the translation of compound annotation between codes characteristic of biology and chemistry.

Taking into account the problems mentioned above, the boundary between bioinformatics and cheminformatics is gradually disappearing as new advances are made in science. The term “bio/cheminformatics” [64] is used in multidisciplinary research in chemistry, biochemistry, and computer science.

## 3. Text-Based Database Screening

Text mining is the most intuitive way of searching for any information on the Internet, including in online databases. The current state of the art in text-based searching in chemical and biomedical sciences was surveyed by Vazquez et al. [65] and Gonzalez et al. [66]. The solutions applied in biomedicine are appropriate for food and nutrition sciences. A common concern in biomedical and food science is health-promoting foods and the risks associated with foods (microbial contamination, allergies, or toxicity).

The first database screening option is based on the name of the compound. The common name or the systematic name can be used. The main disadvantage of this option is that the same compound may have more than one common name. Systematic (chemical) names recommended by the International Union of Pure and Applied Chemistry (IUPAC) are more accurate. They are unambiguous, and they have been developed based on well-defined rules to reflect the compound’s structure [65]. In chemical databases, compound names are presented in English. Users who screen databases based on text-searching options should be familiar with English terminology. Some databases, such as ChemSpider [27], also provide systematic names in other languages (mostly German and French). Linguistic differences should not be a problem for scientists, but they could pose an obstacle for students who are not native speakers of English, do not study chemistry, and are not familiar with English chemical terminology. An additional advantage of systematic names over common names is that they are similar in many languages.

Multilingual websites may serve as dictionaries of chemical terminology. Wikipedia is probably the best known example, and its role in chemical education has recently been emphasized by Walker and Li [50]. It should be stressed that Wikipedia provides cross-links between websites describing the same compound in different languages. The English edition of Wikipedia also contains direct links to databases such as ChemSpider [27] and PubChem [43]. According to Ertl et al. [67], the greatest weakness of Wikipedia is that it contains information about only the most popular compounds, which account for less than 1% of all known compounds. Online translation tools are generally not useful for translating specialist chemical or biological terminology, but some applications, such as Google Translate™, can be modified and updated by users.

Identification numbers in databases, such as PubChem, serve as unambiguous identifiers. The use of ID numbers together with compound names is recommended by research journals (including the International Journal of Molecular Sciences). In some databases, identifiers can be found by translating a compound’s systematic name or chemical code (SMILES or InChI) with the use of the Chemical Translation Service (University of California, Davis, CA, USA) [26]. Chemical names can also be translated into other identifiers in the Chemical Identifier Resolver (National Institute of Cancer, Bethesda, MD, USA) [24]. Other terms associated with compounds may also be used as search queries. The terms and definitions associated with chemical compound classes may be found in the work of Moss et al. [68] and in the IUPAC Nomenclature Database (International Union of Pure and Applied Chemistry—IUPAC).

Biological and medical sciences rely on various ontologies [69]. A univocal definition of the term “ontology” does not exist [69], but it is generally understood as a set of systematically classified keywords for data mining in literature and databases. According to Hoehndorf and co-workers [69], the role of ontologies in biological and medical sciences is to: provide standard identifiers for the classification of phenomena and their relations within a domain (in this case, chemistry, biochemistry and food science), provide a standardized vocabulary for a domain, provide metadata describing the intended meaning of the terms and their relations, and provide machine-readable axioms and definitions enabling computer-aided access and processing.

The ontologies associated with chemical compounds may be found in special databases, such as ChEBI (European Institute of Bioinformatics, Hinxton, UK) [22] and OlsVis (Norwegian University of Science and Technology, Trondheim, Norway) [38]. The National Center for Biotechnology Information (NCBI) (Bethesda, MD, USA) has developed the Medical Subject Headings (MeSH) database [36] which is used in other databases, including PubChem [43].

## 4. Database Search Based on Molecular Structure

Structure-based searching is an attractive alternative to text-based searching. Its popularity is on the rise due to the proliferation of molecule editing programs [70]. Molecule editors are being included in specialized databases by default, and they are also introduced to other websites, such as the English edition of Wikipedia [67].

**Figure 2:**
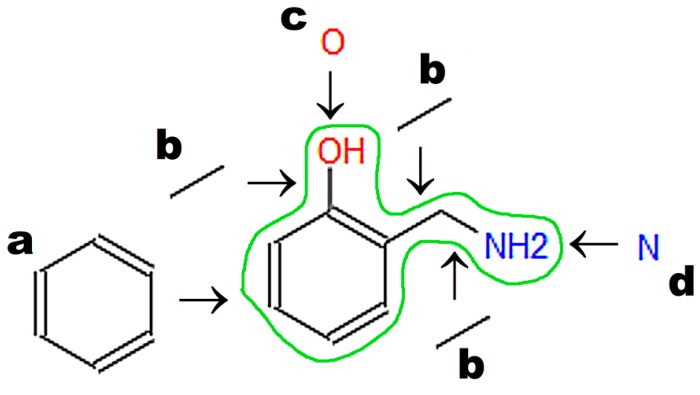
Graphic representation of molecular structure developed in the JSME editor (Basel, Swizerland) [74], which is available on the Chemical Structure Lookup website (National Institute of Cancer, Bethesda, MD, USA) [25]. The letters denote basic primitives: (**a**) benzene ring; (**b**) single bond; (**c**) oxygen atom; (**d**) nitrogen atom.

A graphic representation of a molecular structure developed in a molecule editor is presented in Figure 2. Database users obviously require knowledge of molecular structure, which should be drawn using small, reproducible fragments known as basic primitives [71]. Chemistry students require basic training in molecular editors, in particular if chemistry textbooks in primary and secondary schools do not represent molecular structures built from basic primitives [15]. Special attention should be paid to the graphical representation of stereoisomers [72]. Molecule editors may be used to develop figures for publications and presentations. Programs fulfilling the rules recommended by IUPAC, as listed by Brecher [72], are sufficient for this purpose. Problems associated with graphics quality have been discussed by Clark [73], who enumerates the following crucial factors determining graphics quality: scale (ratio of diagram size to molecule size); font size, line width, bond separation the separation between particular lines presenting multiple bonds, color of all elements of the picture, and precise positioning of particular molecule fragments. Solutions and algorithms recommended to achieve high quality of graphics are also discussed. Clark [73] shows recent and possible future trends in development of new versions of molecular editors.

**Figure 3:**
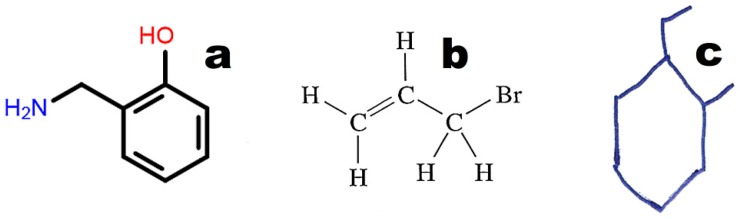
Examples of molecular structures recognized by OSRA: (**a**) structure drawn in a molecular editor using basic primitives; (**b**) classical structure from printed text; (**c**) hand-drawn structure.

The graphical representations of chemical structures can also be converted in the Optical Structure Recognition Application (OSRA) [64]. This program is available on various websites, including the Chemical Structure Lookup [25]. The application recognizes compound structures, including from photographs or scans of printed or even handwritten text. We can recommend structures built from basic primitives. Examples of structures recognized by the OSRA program (National Cancer Institute, Bethesda, MD, USA) are presented in Figure 3.

**Figure 4:**
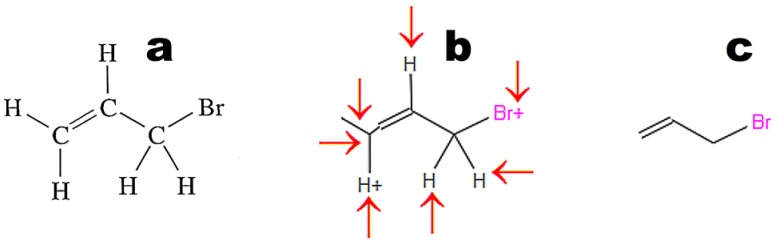
Molecular structure converted by OSRA (structure b in Figure 3): (**a**) original structure; (**b**) structure recognized by OSRA and displayed in a molecule editor (arrows indicate fragments that require curation); (**c**) structure after curation in a molecule editor.

Images converted in OSRA should contain only the chemical structure. The application easily recognizes structures drawn with the use of basic primitives (Figure 3a). The structures shown in Figure 3b,c require curation. Figure 4 illustrates the weak point of OSRA. This application poorly recognizes classic structures displaying all hydrogen atoms. The steps of the recognition process in OSRA and curation in the JSME editor are presented in Figure 4. Structures “a” and “c” in Figure 4 are equivalent. The structure presented in Figure 3c was recognized by OSRA with one error (false positive recognition of one asymmetric carbon atom).

The second option for screening chemical databases based on the molecular structure of a compound involves machine-readable codes such as SMILES, InChI, or InChIKey. SMILES [61] is the most popular machine-readable code, and it is used by default to annotate structures in chemical, biochemical, and medical databases of small molecules. SMILES also has several disadvantages. One molecule can have more than one annotation in SMILES. For instance, 2-(Aminomethyl)phenol (Figure 3) may be annotated as C1=CC=C(C(=C1)CN)O in the Kekule version (used in the PubChem database; CID 70267), c1ccc(c(c1)CN)O in the aromatic version: (used in the ChemSpider database; ID 63452) or NCc1ccccc1O (used by Chemical Structure Lookup). The above representations differ in aromatic ring annotation (double bonds vs. small letters) and/or the sequence of symbols (beginning from carbon or nitrogen). The SMILES code requires special search engines that use all possible representations of a given molecule. InChI and INChIKey [62] generate unique codes for every molecule, which is a key advantage over SMILES. InChI and INChIKey codes may be used as queries in popular text-based search engines (e.g., Google™, Mountain View, CA, USA). InChIKey codes always contain 27 characters and are recommended for text-based searches [75,76]. Information about a compound may be found in online chemical databases as well as other uploaded resources. On the other hand, the results of a Google search based on InChIKeys may be incomplete. Some databases or texts may be missed.

Structure search options in databases include an exact match and similarity search. Compounds whose activity is similar to that of known compounds can be searched in sets of molecules with similar structure. It should be noted, however, that even molecules with a high degree of structural similarity do not always display the same biological activity [77,78]. The determination of the structure–activity relationship and molecule fragments that are crucial for bioactivity may require more detailed analyses.

Similar molecules contain the same functional groups and have similar carbon skeletons. Similarity may be expressed quantitatively. The Tanimoto coefficient is the most popular measure used for this purpose [79,80,81].

The most common notation for the Tanimoto coefficient [79,81] of similarity between molecules 1 and 2 is presented below:

*S*_Tan_ = *c*/(*r* + *d* − *c*) (1)

*r*—number of structural fragments in molecule 1
*d*—number of structural fragments in molecule 2
*c*—number of structural fragments in both molecules

The results of a search based on quantitative similarity may differ depending on database content and the algorithm used for similarity calculation. Differences between algorithms may concern the definition of structural fragments in Equation (1). They may be defined as single bonds, double bonds, or rings, but also as individual atoms with neighborhoods [80]. There are also alternatives to the Tanimoto coefficient [79,80,81]. Special programs for performing similarity searches in major databases according to various specific criteria are available on the website of the University of Bern [49].

Similar molecules can also be searched with the use of the substructure search option which identifies compounds containing the queried molecular structure or its user-defined fragment (e.g., a benzene ring with substituents). The compounds identified by the substructure search option differ from those found in a similarity search. The second option yields compounds with similar molecular mass, whereas the first identifies larger molecules, which usually contain additional substituents instead of hydrogen atoms.

## 5. Examples of Small Molecule Databases

There are a wide variety of small molecule databases that can be subdivided into general and specialized resources. General databases contain information about chemical compounds that belong to various classes and have different biological activities and applications. Specialized databases provide information about narrower classes of compounds, such as food compounds and tasteful molecules. Small molecule databases classified according to structure, biological activity, or application are listed in metabases such as OmicTools (provided by international group) [39], MetaComBio [15], or LabWorm (Jerusalem, Israel).

ChemSpider is a database of the Royal Society of Chemistry (London, UK) [27]. It lists compounds by their systematic names, synonyms (multilingual), structure (2D and 3D images), identifiers (SMILES, InChI and InChIKey), physical and chemical properties, Nuclear Magnetic Resonance (NMR) spectra, as well as references. ChemSpider also provides information about a compound’s compliance with the Rule of five [82] or its violations. The Rule of five was proposed as a criterion for preliminary selection of drug candidates. This application could be expanded to search for potentially bioactive food components. According to the Rule of five, a compound molecule cannot violate more than one of the following criteria: molecular mass less than 500 Da, no more than five hydrogen bond donors, no more than 10 hydrogen bond acceptors, and logarithm of the octanol/water partition coefficient not greater than 5 (measure of hydrophobicity). Information about the experimentally recognized biological activity of compounds is available via external links to other databases (e.g., PubChem, ChEBI, ChEMBL, HMDB, KEGG, FooDB). Moreover, ChemSpider contains links to more than 500 resources and therefore can be regarded as a metabase. One of the greatest advantages of ChemSpider is deep integration [7] with other databases, namely the presence of links to data of individual compounds found by its search engine. ChemSpider offers a standard set of search options, including text searching based on a compound’s name, structure searching in the molecule editor based on identifiers (SMILES, InChI, or InChIKey), and substructure and similarity searching. Compounds can be queried in ChemSpider via Google™ based on an InChIKey string. Links to compound data in ChemSpider are also available in Wikipedia (English edition) and the Chemical Structure Lookup Service.

The presence of a user-friendly, structure-based search engine and external links to many databases may be considered as the major strong points of ChemSpider. In some cases the databases are easier to access via ChemSpider than via their own search engines. This advantage is, however, not visible enough on the database website. Users looking for information concerning the biological activity of a compound should find a compound card tab named “More” and then click “Data sources”. Other advantages of ChemSpider are the high quality of the graphics and the possibility of Google™ searching via a simple click on InChI or InChIKey. The database is continuously updated. Updates include addition of new compounds and new resources.

The PubChem database [43] is operated by the National Center for Biotechnology Information (NCBI) in Bethesda, MD, USA. It contains comprehensive information about compounds and substances (mixtures of compounds, solutions, extracts). Every listed item has a unique compound identifier (CID) or substance identifier (SID). The following information is provided for every compound: structure, chemical identifiers, synonyms, and physicochemical properties (experimentally found and predicted). PubChem contains important data for biological and medical research, including biological test results, pharmacology, toxicity, and references. In contrast to ChemSpider, all resources are available on the PubChem website. The database offers a standard set of text-based search and structure-based search options, including structure searching in a molecule editor, queries based on SMILES or InChI strings, and exact match, similarity, substructure, or superstructure searching. PubChem is the largest and most popular database of experimentally investigated small molecules, with around 89 million compounds and 220 million substances (as of May 2016). It is a reference database for other resources. Compound data in PubChem can be accessed from other databases, including ChemSpider, ChEMBL, FooDB, KEGG, and BRENDA. The English edition of Wikipedia contains hyperlinks to compound data in PubChem, and it lists the CIDs of particular compounds in other languages. PubChem may be recommended for educational purposes as the first database to be learned [15].

PubChem is to date the biggest database of experimentally discovered and synthesized chemical compounds with small molecular weight. The database is continuously updated. Another advantage of this database is organization of data concerning individual compound at the website. Comprehensive data may be accessed via a single tab. The PubChem contains results of biological tests even if they did not resulted in detection of bioactivity. The database includes both chemical (SMILES, InChI, InChIKey) and biological descriptors of compounds (e.g., amino acid sequences of peptides or graphic symbols for oligosaccharides). This policy is a major advantage in light of the problems of communication between biological and chemical sciences, reported in Section 2. A lack of links to other databases may be considered the weak point of PubChem. Sometimes other databases contain data about compound bioactivity, absent in PubChem. The PubChem search engine is not as simple as those of ChemSpider or ChEMBL.

The ChEMBL database [23] is operated by the European Bioinformatics Institute (EBI), Hinxton, UK. Standard compound data includes 3D structure, names and identifiers (SMILES, INChI, InChIKey), chemical and physical properties, including Rule of Five violations, information about biological activity, and links to compound information in other databases such as PubChem and KEGG. Unlike PubChem, ChEMBL contains comprehensive information about small molecules as well as the interacting target proteins, such as enzymes inhibited by compounds annotated in the database. ChEMBL offers standard search options, including text-based search based on a compound’s name and structure searching based on chemical codes or structures developed in the molecule editor (exact match, similarity, or substructure search). Proteins interacting with small molecules may be queried by name or sequence similarity. ChEMBL entries are accessible via Wikipedia (especially the English edition) and Google search using InChIKeys.

The ChEMBL contains information about ca. 2 million compounds and is continuously updated. The major strength of ChEMBL as compared with ChemSpider and PubChem is the presence of information about proteins interacting with small molecules. The search engine is designed to meet the expectations of users interested in biological activity. Possible queries include target proteins and assays. The search engine of ChEMBL is user-friendly. The set of compounds annotated in ChEMBL is smaller than that in PubChem. This can be considered as a minor weak point. In contrast to the second database, ChEMBL contains links to compound data in few databases. Links to UniProt and KEGG databases provide useful information about target proteins and metabolism of small molecules, respectively.

Specialized databases are of particular interest for food scientists. Examples of specialized databases include FooDB, which lists food components, and SuperSweet [46], which contains information about sweet substances. FooDB is provided by WishartLab at the University of Alberta, Edmonton, Canada. It contains information about molecular structure, physicochemical properties, biological activity, MS and NMR spectra, compound distribution in foods, and links to other databases (e.g., PubChem and HMDB). Text-based search options rely on the names of compounds and organisms that are sources of food (English and Latin names of organisms). Typical structure search options are also available.

The strength of the FooDB database is the possibility of screening MS/MS and NMR spectral libraries using a user’s experimental data as the query. This option makes it a useful tool for “omics” analyses. FooDB may be a useful tool for diet design and calculation of intake of particular bioactive compounds due to the presence of quantitative data concerning their content in various foods of animal and plant origin. FooDB contains data concerning 26,630 compounds from 907 food resources.

SuperSweet, a database provided by Charité—Universitätsmedizin Berlin, Germany, lists natural and synthetic sweet substances [46]. Compound information includes synonyms, sweetness (quantified as the ratio of compound sweetness to glucose sweetness), visualization of molecular docking to a sweetness receptor model, and links to PubChem. Search options include property searching based on a compound’s name (text-based search), compound class, molecular characteristics (mass, number of atoms, number of rings), as well as structure searching via a molecule editor and similarity searching.

The SuperSweet database provides the usual information available in PubChem with the exception of sweetness value and visualization of docking to sweet receptor for part of compounds. There are no links to databases compatible to PubChem. SuperSweet is an example of a database facilitating access to data concerning a narrow group of compounds (in this case, sweet ones) as compared with more general databases.

## 6. Examples of Databases of Enzymes, Metabolites, and Metabolic Pathways

Enzymatic reactions play an increasingly important role in food technology [46]. Information from databases may support investigations aimed at application of enzymes in food technology. Predicting reactions that produce a compound of interest and/or reactions where the compound is used as a substrate constitute important challenges for cheminformatics in the area of enzymology [83]; some information annotated in databases is applicable in food and nutrition science. Food technologists designing a process involving enzymatic reactions may ask many questions. Which enzyme is sufficient for our process? Which organism may be used as the source of the enzyme? How can we produce and purify the enzyme? What are the optimal conditions for the reaction (temperature, pH)? Taking into account the fact that many enzymes catalyze more than one reaction [84], we can ask the following questions. What byproducts may appear as a result of our enzymatic process? How might these compounds affect product quality (e.g., taste and flavor)? Nutritionists are interested in the contribution of particular compounds in enzymatic reactions in humans. The question to be asked by nutritionists is, for instance: “What is the contribution of our compound of interest in enzymatic reactions occurring in humans”? Databases summarizing data on enzymes may facilitate access to information concerning the questions mentioned above [85]. There are general databases of enzymes and their ligands, such as ExplorEnz [30] and BRENDA [20]. Examples of databases listing specific groups of enzymes include e.g., CAZy (Architecture et Fonction des Macromolécules Biologiques—AFMB, Marseille, France), a database of enzymes catalyzing reactions of carbohydrates [21], and MEROPS (The Wellcome Trust Sanger Institute, Hinxton, UK), a database of proteolytic enzymes [35].

Another possible question concerns metabolism. A typical nutritionist’s question is “How does my compound of interest affect human metabolism”? Technologists may be interested in the influence of food components or contaminants on the metabolism of microorganisms involved in technological processes (such as cheesemaking). Information relevant for this area of interest is available in databases of metabolites providing information about the localization and role of different compounds in the network of metabolic pathways [86,87,88].

Contemporary experimental work in the areas of food and nutrition science is significantly enhanced by the introduction of metabolomics strategies involving simultaneous analysis of many compounds [89]. Mass spectrometry and nuclear magnetic resonance are basic methods used in such analyses. Databases of metabolites are also designed for support metabolomics analyses e.g., by annotation of reference mass and NMR spectra. Databases and other bioinformatics and cheminformatics tools that are used in mass spectrometry-based metabolomics experiments have been recently reviewed by Misra and van der Hooft [90] and Vinaixa and co-workers [91]. Bioinformatics tools are also available for experiments involving nuclear magnetic resonance techniques [90,92].

The databases are described in this section in the following order: database focused solely on enzymes and serving as an introductory resource to other databases—ExplorEnz [30]; database summarizing comprehensive data about enzymes and their ligands—BRENDA [20]; database linking data about compounds, enzymes, and metabolic pathways—KEGG [33]; and exemplary database containing information supporting experiments involving a metabolomics approach—HMDB [34].

ExplorEnz [30] was developed by Trinity College, Dublin, Ireland, and it may be recommended as the first option for searching enzymes based on their number or name. This database may serve as a guide to enzyme classification. ExplorEnz lists enzymes classified by the International Union of Biochemistry and Molecular Biology (IUBMB) according to their enzyme classification (EC) number. The database contains brief information about enzyme specificity with links to more comprehensive resources, such as BRENDA or KEGG. ExplorEnz may be regarded as a metabase, which is integrated at the level of particular entries. Text-based searching options are available based on an enzyme’s name or number. Users can also browse the complete list of enzymes and perform a manual search.

ExplorEnz, due to its simplicity, may be recommended as the first option for finding information about enzymes. On the other hand, details may be found only via external links. ExplorEnz may thus be considered as an enzyme metabase. The advantage of ExplorEnz is the visibility of external links (in contrast to e.g., ChemSpider, Royal Society of Chemistry, London, UK).

The BRENDA database [20] is operated by the Technical University in Braunschweig, Germany. This resource lists compounds, enzymes, and metabolic pathways. Compound information includes name, structure, InChIKey, role as enzyme ligand (substrate, product, inhibitor, activator, or cofactor), kinetic data of enzymatic reactions involving the compound, references and links to compound data in PubChem, and ontologies relating to the queried compound in ChEBI. Enzyme information includes name, EC number, catalyzed reaction, reaction type, links to information about the pathway involving the searched enzyme, enzyme structure, molecular properties (cloning, purification details, engineering and application), diseases associated with inappropriate activity of the enzyme, references, and links to other databases. Search options include text-based searching (possible queries: compound name, enzyme name, enzyme number), substructure searching (involving structures input via a molecule editor), sequence searching (amino acid sequence), genome explorer (search option based on the taxonomy of organisms synthesizing enzymes), functional enzyme parameters, and enzyme reactions (BKM react online). BRENDA can be screened via Google™ with InChIKey as the query. Enzyme data may be also accessed via ExplorEnz or KEGG databases.

The strong point of BRENDA is that this database contains the most comprehensive information about individual enzymes. This information includes optimal conditions for enzyme action, organisms producing the enzyme, references concerning cloning, and purification methods. Such information may be sufficient for food technologists and biotechnologists. Information about enzyme ligands seems to be complementary to that available in PubChem or ChEMBL. Some annotations concerning the inhibition of enzymes by low-molecular compounds are available only in BRENDA. Other advantages of this database are more search options than other enzyme databases and the quality of graphic schemes visualizing metabolic pathways. On the other hand, users who need a compact summary of a given enzyme can find it in KEGG or ExplorEnz.

The Kyoto Encyclopedia of Genes and Genomes (KEGG) [33] was developed by Kyoto University, Japan. It contains comprehensive information about the functions of living organisms at all levels: genomes, genes, proteins, enzymes, metabolic pathways, and small molecules. A detailed presentation of the entire content of KEGG exceeds the scope of this review. Selected sections of the database are dedicated to small molecules (KEGG COMPOUND), reactions catalyzed by enzymes (KEGG REACTION), and maps of metabolic pathways (KEGG PATHWAY). The database also contains information about specific compound groups (carbohydrates, peptides, and lipids). Tabs in KEGG are cross-linked. Compound data are linked with the associated enzymes and pathways, and enzyme data are linked with the relevant compounds and pathways. Information about metabolic pathway includes links to the associated enzymes and compounds. KEGG also provides links to other databases containing information about compound properties (PubChem), ontologies associated with a given compound (ChEBI), and enzyme data (ExplorEnz and BRENDA). The resources in KEGG can be accessed via other databases, including ChemSpider (compound data) and ExplorEnz (enzyme data).

The strong point of KEGG is its architecture enabling finding information about a compound, its reactions, enzymes catalyzing these reactions, and metabolic pathways via the network of crosslinks. The search engine providing only a text search opportunity seems to be a weakness of this database.

BRENDA and KEGG may be used as complementary resources. KEGG contains general information, and it facilitates navigation between databases of enzyme-catalyzed reactions, their substrates, products, and metabolic pathways. BRENDA is a source of more detailed data, in particular in relation to enzymes and their ligands. For instance, the activators and inhibitors of a specific enzyme are listed in BRENDA. KEGG may be used to search for basic information about an enzyme, whereas BRENDA contains detailed data relating to the optimal conditions for enzyme activity (pH and temperature), with comprehensive references. BRENDA and KEGG are cross-referenced with other databases, including the HMDB database of metabolites, specialized databases such as the MEROPS database of proteolytic enzymes [35] or the CAZy database of carbohydrate-active enzymes [21]. BRENDA and KEGG may be accessed from CAZy via the ExplorEnz database.

The Human Metabolome Database (HMDB) [32] is kept by the University of Alberta, Edmonton, Canada. The database may be regarded as complementary to BRENDA and KEGG because it focuses on metabolite properties, which are important in experiments involving MS and NMR methods. The HMDB contains information about compounds that have been detected and/or determined in the human metabolome (name, structure annotated with the use of chemical codes, ontologies, physical properties, information about MS and NMR spectra, concentration in biological fluids, references, and links to compound information in other databases, including PubChem, ChemSpider, FooDB, and KEGG). Links to compound data in FooDB are particularly useful for research in the field of nutrition. The database also contains information about reactions involving the queried metabolites, enzymes catalyzing these reactions, metabolic pathways, biofluids containing compounds of interest, and diseases associated with abnormal concentration and metabolism of particular substances. Compounds are classified based on their status (detected or quantified) and the presence in specific biofluids (e.g., serum, milk, and blood). Standard search options include text-based searching, structure searching (including structures input via the molecule editor), and sequence searching (enzymes). Items can also be searched based on mass spectrometry or nuclear magnetic resonance data. The following options are available: mass spectrometry (MS), tandem mass spectrometry (MS/MS), gas chromatography-mass spectrometry (GC-MS), one-dimensional nuclear magnetic resonance (1D-NMR), and two-dimensional nuclear magnetic resonance (2D-NMR). Compound data in HMDB may also be accessed via links in ChemSpider or FooDB.

The HMDB database contains recent data concerning ca. 42,000 metabolites. Opportunities for support metabolomics studies performed using mass spectrometry or NMR appears to be a major strong point of the database. Search options mentioned above are supported by the possibility of browsing data concerning compounds, their reactions and pathways, biofluids, etc. More recent versions of the database (version 3.6 is the most recent in November 2016) are enriched as compared with previous ones. They contain more metabolites and more information about individual compounds.

## 7. Navigating the Network of Links and Cross-Links between Databases

**Figure 5:**
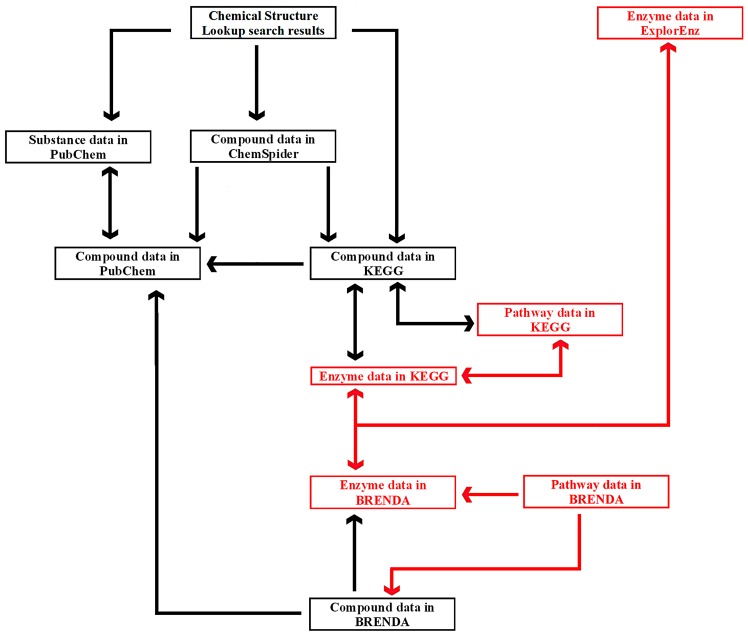
Diagram of links optionally redirecting the user to information about specific compounds, enzymes, and metabolic pathways in the discussed databases. Compound data and the relevant links are marked in black, and enzyme and pathway data with links are in red. Arrows indicate possible directions of search.

The links between six exemplary tools—Chemical Structure Lookup, ChemSpider, PubChem, KEGG, BRENDA, and ExplorEnz—at the level of individual compounds, enzymes, and metabolic pathways are presented in Figure 5. A similar diagram is used by students enrolled in the Enzymology, Bioinformatics, and Bioprocesses course as part of the Food Engineering specialty program, which is held by the University of Warmia and Mazury in Olsztyn, Poland, in collaboration with the University of Applied Sciences in Offenburg, Germany.

A database can be searched with any of the tools presented in Figure 5. Compound data can be found directly in PubChem as well as other resources. Chemical Structure Lookup and ChemSpider provide access to many databases that are not listed in the diagram. Comprehensive information about a compound’s biological activity may not be available from a single database. For instance, a peptide with the sequence DA (Asp-Ala) is an inhibitor of three proteolytic enzymes: angiotensin I-converting enzyme (EC 3.4.15.1), dipeptidyl-peptidase III (EC 3.4.14.4), and glutamate carboxypeptidase II (EC 3.4.17.21). Information about the peptide’s inhibitory effect on the first enzyme is presented in PubChem (CID: 5491963) and ChEMBL (not shown in the diagram; ID: CHEMBL17503), whereas data relating to the second and third enzymes can be found in BRENDA (Ligand: Asp-Ala). For this reason, complementary databases should be screened to provide the most comprehensive information. Metabases such as Chemical Structure Lookup or ChemSpider provide access to information about specific compounds in multiple databases and catalogs. Users can choose the preferred source of data. Chemical Structure Lookup contains direct links to compound data in ChemSpider, but reverse links (ChemSpider to Chemical Structure Lookup) are not yet available (November 2016).

The diagram emphasizes the difference in the status of PubChem and ChemSpider. PubChem is a reference database. In Figure 5, arrows from other tools lead to compound data in PubChem, which is linked with ChemSpider, BRENDA, KEGG, and many other databases. Users can find enzyme data in ExplorEnz, links to information about that enzyme and compounds that act its ligands in BRENDA or KEGG, and links to data concerning the same compounds in PubChem. The route of the query in the network of cross-linked databases indicates that ChemSpider could be a bifurcation site. Compound data is linked with the relevant resources in PubChem, KEGG, and many other databases. KEGG and BRENDA provide users with comprehensive information about a queried compound by linking references to small molecules and biomacromolecules. The tools presented in Figure 5, such as Chemical Structure Lookup, ChemSpider, KEGG, and ExplorEnz (other sites of bifurcation in the network) contain links to numerous websites not included in the diagram. Users can begin the search by querying a compound in ChemSpider, finding a link to compound data in KEGG, and, subsequently, to information about reactions where the queried compound is a substrate or a product, enzymes that catalyze those reactions, and metabolic pathways that involve the searched compounds and enzymes. A dedicated search engine has recently been implemented in BRENDA (June 2016). The network of cross-links between databases also contains gaps. In Figure 5, such a gap is represented by an absence of links between compound data in ChemSpider and BRENDA (as of November 2016). Such links would be useful due to the fact that BRENDA contains information about compound activities not available in PubChem, ChEMBL, and other databases linked from ChemSpider. Reverse links (from BRENDA to ChemSpider) would facilitate access to multiple databases annotating information about compounds of interest.

**Figure 6:**
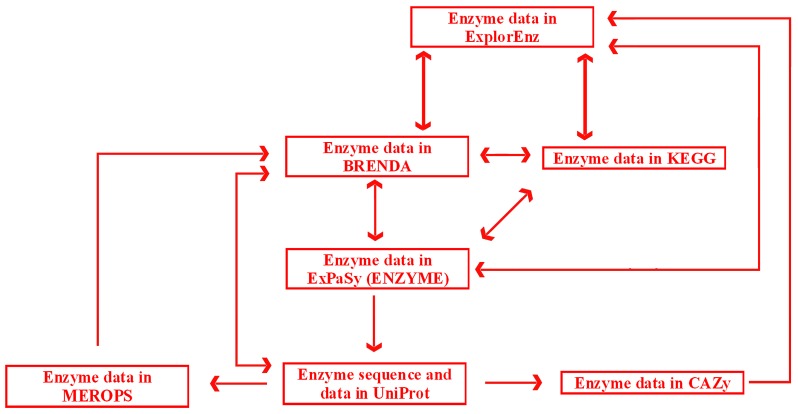
Diagram of links between enzyme data in exemplary databases. Arrows indicate possible directions of search. The color coding scheme is the same as in Figure 5.

Links and cross-links between exemplary, general, and specialized databases of enzymes are presented in Figure 6. A search may be initiated in any database with the use of a dedicated search engine. ExplorEnz acts as a metabase by providing direct links to enzyme data in BRENDA, KEGG, ENZYME, and other databases not indicated in Figure 6. Specialized databases dedicated to a specific category of enzymes, such as proteolytic enzymes (MEROPS) or enzymes catalyzing carbohydrate reactions (CAZy), provide links to more general databases (BRENDA or ExplorEnz). Reverse links are often not available (as of July 2016). BRENDA does not provide direct links to data of particular enzymes in MEROPS. Such links would be useful due to the fact that MEROPS contains comprehensive information concerning the specificity of enzymes, not available in BRENDA. The information queried in BRENDA has to be searched via other databases such as the UniProt protein sequence database. UniProt (UniProt Consortium, UK, Switzerland, USA) contains links to enzyme data in MEROPS. Links from MEROPS to other databases, such as ExplorEnz or KEGG, are available via BRENDA. CAZy contains links to other databases via ExplorEnz, and can be accessed via direct links in UniProt.

Practical applications of the network of databases have been discussed in our previous publication [19]. The BIOPEP (University of Warmia and Mazury in Olsztyn, Poland) database of sensory peptides and amino acids is built, enriched, and updated by screening of other databases to find information about the biological activity of specific compounds. Sensory peptides act as inhibitors of enzymes, in particular proteolytic enzymes [93]. Amino acid sequences in single-letter code are translated into SMILES and other chemical codes with the use of a previously described procedure [19]. Peptide structures annotated in SMILES are used as queries in the ChemSpider database. Compound data in ChemSpider includes direct links to data in PubChem, ChEMBL, and other databases containing information about the bioactivity of compounds (Figure 5). In BRENDA [20], compounds are queried independently based on sequences in three-letter code. Specialized databases of peptides, such as the BIOPEP database of bioactive peptides [94] or SATPdb (Insitute of Microbial Technology, Chandigargh, India) [44], are also used. SATPdb is a metabase with links to peptide data in other databases, such as the AHTPDB (Insitute of Microbial Technology, Chandigargh, India) [18] database of peptides inhibiting angiotensin I-converting enzyme (EC 3.4.15.1).

## 8. Application of Databases in Analyses of Datasets and Interpretation of Experimental Results in Food and Nutrition Sciences

A secondary analysis of datasets is performed on data collected by a third party for another primary purpose [9]. The time and cost of research studies that rely on this strategy is significantly reduced in comparison with most studies that involve primary (experimental) data collection. Online databases provide comprehensive information, and they are commonly used in research [9]. Several applications of secondary analysis of datasets in studies investigating the metabolism and biological activity of low-molecular-weight food components are presented below.

Medina-Franco and co-workers [95] analyzed the similarities in the structure and physicochemical properties of GRAS (Generally Recognized as Safe) food ingredients listed by FEMA (Flavor and Extract Manufacturers Association), drugs and natural products found in databases such as DrugBank (University of Alberta, Edmonton, AB, Canada) [23], AnalytiCon (AnalytiCon Discovery, Potsdam, Germany), and Specs (Specs, Zoetermeer, The Netherlands). The space of GRAS food ingredients partially overlapped a broad region of the space occupied by the structural and physicochemical properties of drugs. The lipophilicity profile of GRAS compounds, a key property in predictions of bioavailability for humans, was particularly similar to that of the approved drugs. The above approach was expanded [96] by searching for information about GRAS compounds in other databases, including the SuperScent database of flavor compounds (Charité—Universitätsmedizin Berlin, Germany) [44] and the TCM database (Laboratory of Computational and Systems Biology, China Medical University, Taichung, Taiwan) of natural products used in traditional Chinese medicine [47]. The results of the analysis revealed that the structure and physicochemical properties of selected flavor-enhancing ingredients are similar to those of analgesics and satiety agents.

The similarities between the structure and properties of FEMA GRAS food ingredients and mood stabilizing drugs were studied in silico by Martínez-Mayorga and co-workers [97]. Selected compounds were highly similar to valproic acid (PubChem CID: 3121; ChemSpider ID 3009), an inhibitor of histone deacetylase (EC 3.5.1.98) that is targeted by mood stabilizers. Two food ingredients, nonanoic acid (PubChem CID 8158; ChemSpider ID 7866) and (2E)-decenoic acid (PubChem CID: 94282; ChemSpider ID 4445851), with similar molecular structure to valproic acid, inhibited histone deacetylase in vitro. The results of in silico predictions were confirmed experimentally.

These in silico studies compared the chemical space of flavor-enhancing components and drugs. Conclusions about the possible bioactivity of food ingredients were drawn based on overlapping chemical spaces, defined as ensembles of all organic molecules to be considered when searching for new bioactive compounds [98].

Boto-Ordóñez and co-workers [99] relied on the Phenol-Explorer database (University of Alberta, Edmonton, AB, Canada) [41] to predict the metabolism of phenolic compounds from various types of wine. They developed a metabolic pathway map of ingested wine compounds. The map included both qualitative (identification of metabolites) and quantitative (concentration in wines and in body fluids) aspects. The results expand our understanding of the health-promoting effects of phenolic compounds from wine.

Ganesan and Brown [100] performed an in silico study aiming to predict the influence of sodium replacement by potassium or calcium on cheese flavor. Cheese flavor is affected by low-molecular-weight metabolites of bacterial strains used in cheese ripening. The replacement of sodium by other cations influenced the kinetics of enzymes involved in 135 bacterial metabolic pathways. Bacterial enzymes and pathways affected by metal cations are described in the ProCyc database (Utah State University, Logan, UT, USA) [36,42]. The results of the cited study are interesting from the point of view of production and consumer acceptance of cheeses with low sodium content.

Possible interactions between 4000 food components and drugs were investigated in silico by Jensen and co-workers [101]. The human diet may deliver positive or negative effects when combined with specific drug treatments. Specific food components may enhance or suppress a drug’s efficacy by influencing pharmacokinetics (absorption, distribution, metabolism, and excretion of drugs) and pharmacodynamics, processes that are related to a drug’s mechanisms of action. Food and drug components may interact with the same target, such as an enzyme. Food components may also affect the activity of enzymes involved in drug metabolism. The relevant information is available in the NutriChem database (Technical University of Denmark, Lyngby, Denmark) [37].

Databases are also used in experimental work. Ridder and co-workers [102] relied on information about green tea compounds and their possible reactions to predict a set of metabolites. The dataset was used to identify products of green tea metabolism in human urine by mass spectrometry. The authors compared their dataset with the PubChem database. They predicted ca. 27,000 possible metabolites resulting from reactions of 75 tea compounds. Only 23% of these compounds were present in PubChem. Some of the experimentally identified metabolites were predicted, but not present in this database. These results show that even the most extensive database of chemical compounds is still far from complete.

Suh et al. [103] used the HMDB database [32] to confirm compounds identified in a study of changes in the metabolites of a mixture of *Cudrania tricuspidata*, *Lonicera caerulea*, and soybeans. The metabolites were identified and quantified by mass spectrometry. Their anti-obesity effect was also studied in vivo (in mice). The aim of the experiment was to determine the influence of fermentation on the anti-obesity effect of products made from the analyzed plants.

Databases may serve for calculation of intake of particular food components. Examples of such database applications have been described by Witkowska and co-workers [104]. They calculated the daily intake of phenolic compounds using data concerning their content in various foods, annotated in PhenolExplorer [41] and USDA (United States Department of Agriculture, Wshington, DC, USA) databases.

In the cited research studies, hundreds or even thousands of compounds and their reactions were analyzed in silico. Databases significantly facilitated researchers’ work and supported the compilation of datasets within a reasonable time. Database curation standards are being constantly improved [105,106,107] to overcome the limitations associated with these tools [84] and make the tasks mentioned above easier. In the Era of Big Data, humans generate enormous amounts of information, which may be very difficult to find in either the literature [108] and databases. Users can compile resources from more than one database and directly from the literature to build datasets for further research. A dataset developed with the involvement of this strategy were described by Fernández-de Gortari and Medina-Franco [11]. Datasets compiled by researchers from the literature may be made available on the Internet and thereby facilitate related research, including secondary analysis [9]. Supplement to the review about taste of food peptides [93] including data concerning bioactivity, especially the inhibition of proteolytic enzymes, may serve as an example of such a dataset.

## 9. Final Remarks

The growing number of databases of food components provides new research and educational opportunities not only in chemistry and biology, but also in food and nutrition science. In addition to chemical and biochemical data, various types of information about food and nutrition may be annotated and published in databases. Databases offer unprecedented search opportunities for scientists, from the retrieval of information about specific compounds to sophisticated, high-throughput analyses of large datasets.

Databases always contain “second-hand” information and, therefore, have certain limitations. The available information may be incomplete or may contain errors. The process of compiling information and data correction, if necessary, could be significantly accelerated by encouraging database users and authors of published research to submit their findings. Wikipedia is the best proof that this could be a viable solution. Another opportunity to be considered by authors of research involving data analysis is publication of datasets of interest e.g., as supplements to articles describing results.

Databases are expanded to include numerous links and cross-links, thus forming a complex network. This network may be utilized to find more complete information about a compound or set of compounds than in a single database. The visibility of specialized databases can be improved by organizing them into metabases. This advantage is important especially for new databases, which are not well known to start with. We recommend comparison of information from more than one database to receive reliable and comprehensive information. Metabases may facilitate such confrontation and thus are a good starting point for data searching. Users can rely on those tools to compile extensive datasets and avoid errors. Metabases may contain links to homepages of particular tools. MetaComBio, OmicTools, and LabWorm are examples of such metabases. The opportunity for rapid addition of new tools is an advantage of these databases. Building links from a compound or enzyme entry in a metabase to data of the same compound or enzyme in particular databases is an alternative solution. ChemSpider is an example of a compound metabase whereas ExplorEnz is an enzyme metabase providing links to individual entities. Metabases would facilitate finding complementary databases. Two databases annotating the same compound may be understood as complementary if the first one contains information unavailable in the second one and vice versa. Complementary databases may contain, for instance, information from the areas of biology and chemistry, annotated using codes specific to these two approaches. Two databases containing the same information, with the same set of references, may be understood as competitive.

Examples given in this article cover only a small part of the worldwide network of databases. This network grows by the addition of new databases and by the creation of new links and cross-links between existing ones. Future research can be aimed at filling the gaps in the database network, i.e., a lack of links providing value-added information not available via a single database. On the other hand, the quality of databases and datasets increases due to implementation of improved standards of curation. Another expected direction of bio/cheminformatics development is the implementation of a new generation of databases and programs breaking the language barrier between chemistry and the biological sciences (biochemistry and molecular biology), as this remains a challenge. This problem is especially noted in food science because the field takes information and inspiration from both chemistry and biology.

## Figures and Tables

**Table 1:** Summary of databases and programs cited in this publication.

Tool	Address	Category ^1^	Reference
AHTPDB	http://crdd.osdd.net/raghava/ahtpdb/	Amino acids and peptides	[18]
AnalytiCon ^2^	http://www.ac-discovery.com/	-	-
BIOPEP	http://www.uwm.edu.pl/biochemia/index.php/pl/biopep	Amino acids and peptides; Flavor, aroma and taste enhancing compounds	[19]
BRENDA	http://www.brenda-enzymes.org/	Biochemical reactions; Metabolites and metabolic pathways	[20]
CAZy	http://www.cazy.org/Welcome-to-the-Carbohydrate-Active.html	Biochemical reactions	[21]
ChEBI	http://www.ebi.ac.uk/chebi/	Miscellaneous compounds	[22]
ChEMBL	https://www.ebi.ac.uk/chembldb/	Miscellaneous compounds	[23]
Chemical Identifier Resolver	https://cactus.nci.nih.gov/chemical/structure	Programs	[24]
Chemical Structure Lookup	http://cactus.nci.nih.gov/cgi-bin/lookup/search	Metabases, Programs	[25]
Chemical Translation Service	http://cts.fiehnlab.ucdavis.edu/	Programs	[26]
ChemSpider	http://www.chemspider.com/Default.aspx	Miscellaneous compounds, Metabases	[27]
DrugBank	http://www.drugbank.ca/	Pharmacologically active compounds	[28]
ExPASy ENZYME	http://enzyme.expasy.org/	Biochemical reactions	[29]
ExplorEnz	http://www.enzyme-database.org/index.php	Biochemical reactions	[30]
FEMA GRAS	http://www.femaflavor.org/fema-gras%E2%84%A2-flavoring-substance-list	Food components, Flavor aroma and taste affecting components	[31]
FooDB	http://foodb.ca/	Food components	
Google Translate™	https://translate.google.com/	-	
HMDB	http://www.hmdb.ca/	Metabolites and metabolic pathways	[32]
IUPAC Nomenclature Database	http://www.chem.qmul.ac.uk/iupac/	Education	
KEGG	http://www.genome.jp/kegg/	Metabases, Biochemical reactions; Metabolites and metabolic pathways	[33]
LabWorm	https://labworm.com/	Metabases	
LipidMaps	http://www.lipidmaps.org/	Lipids	[34]
MEROPS	http://merops.sanger.ac.uk/	Biochemical reactions	[35]
MeSH	https://www.nlm.nih.gov/mesh/	Miscellaneous compounds	[36]
MetaComBio	http://www.uwm.edu.pl/metachemibio/index.php/about-metacombio	-	[15]
NutriChem	http://www.cbs.dtu.dk/services/NutriChem-1.0/	Food components	[37]
OLSVis	http://ols.wordvis.com/	Miscellaneous compounds	[38]
OmicTools	http://omictools.com/	Metabases	[39]
Open Babel	http://openbabel.org/wiki/Main_Page	-	[40]
PhenolExplorer	http://phenol-explorer.eu/	Food components, Phenolic compounds	[41]
ProCyc	http://procyc.westcent.usu.edu:1555/	Metabolites and metabolic pathways	[42]
PubChem	https://pubchem.ncbi.nlm.nih.gov/	Miscellaneous compounds	[43]
SATPdb	http://crdd.osdd.net/raghava/satpdb/	-	[44]
Specs ^2^	http://www.specs.net/snpage.php?snpageid=home	-	-
SuperScent	http://bioinf-applied.charite.de/superscent/	Flavor-, aroma-, and taste-enhancing compounds	[45]
SuperSweet	http://bioinf-applied.charite.de/sweet/	Flavor-, aroma-, and taste-enhancing compounds	[46]
TCM	http://tcm.cmu.edu.tw/	Pharmacologically active compounds	[47]
UniProt	http://www.uniprot.org/	-	[48]
University of Bern website	http://www.gdb.unibe.ch/	Programs	[49]
USDA	http://fnic.nal.usda.gov/food-composition	Food Components	
Wikipedia	https://en.wikipedia.org/wiki/Main_Page	-	[50]
WURCS	http://www.wurcs-wg.org/software.php	Programs	[51]

^1^ Category according to the MetaComBio website (University of Warmia and Mazury in Olsztyn, Poland) [15]; ^2^ Commercial resource. All tools were accessed between May and November 2016.

## Abbreviations

AHTPDB	Antihypertensive Peptides Database
CAZy	Carbohydrate Active Enzyme Database
CID	Compound Identifier (in PubChem database)
EC	Enzyme Classification
GC-MS	Gas chromatography–mass spectrometry
FEMA	Flavor and Extract Manufacturers Association
GRAS	Generally Recognized As Safe
HMDB	Human Metabolome Database
InChI	International Chemical Identifier
IUBMB	International Union of Biochemistry and Molecular Biology
IUPAC	International Union of Pure and Applied Chemistry
KEGG	Kyoto Encyclopedia of Genes and Genomes
MeSH	Medical Subject Headings
MS	Mass Spectrometry
MS/MS	Tandem Mass Spectrometry
NCBI	National Center for Biotechnology Information
NMR	Nuclear Magnetic Resonance
OSRA	Optical Structure Recognition Application
SATPdb	Structurally Annotated Therapeutic Peptides database
SID	Substance Identifier (in PubChem database)
SMILES	Simplified Molecular Input Line Entry System
TCM	Traditional Chinese Medicine
WURCS	Web Unique Representation of Carbohydrate Structures
